# Machine Learning Based Rank Attack Detection for Smart Hospital Infrastructure

**DOI:** 10.1007/978-3-030-51517-1_3

**Published:** 2020-05-31

**Authors:** Abd Mlak Said, Aymen Yahyaoui, Faicel Yaakoubi, Takoua Abdellatif

**Affiliations:** 8 Digital Research Centre of Sfax, Sfax, Tunisia; 9 Institut Mines-Télécom, CNRS, Paris, France; 10 Université de Sherbrooke, Sherbrooke, QC Canada; 11 Digital Research Centre of Sfax, Sfax, Tunisia; 12 University of Sfax, Sfax, Tunisia; 13 Military Academy of Fondouk Jedid, Nabul, Tunisia; 14 SERCOM Lab, Polytechnic School of Tunisia, La Marsa, Tunisia; 15 Defense Science and Technology Laboratory, Tunis, Tunisia

**Keywords:** Internet of Things, Smart hospitals, Intrusion detection, Rank attack, Machine learning, RPL

## Abstract

In recent years, many technologies were racing to deliver the best service for human being. Emerging Internet of Things (IoT) technologies made birth to the notion of smart infrastructures such as smart grid, smart factories or smart hospitals. These infrastructures rely on interconnected smart devices collecting real-time data in order to improve existing procedures and systems capabilities. A critical issue in smart infrastructures is the information protection which may be more valuable than physical assets. Therefore, it is extremely important to detect and deter any attacks or breath to the network system for information theft. One of these attacks is the rank attack that is carried out by an intruder node in order to attract legitimate traffic to it, then steal personal data of different persons (both patients and staffs in hospitals). In this paper, we propose an anomaly based rank attack detection system against an IoT network using Support Vector Machines. As a use case, we are interested in the healthcare sector and in particular in smart hospitals which are multifaceted with many challenges such as service resilience, assets interoperability and sensitive information protection. The proposed intrusion detection system (IDS) is implemented and evaluated using Conticki Cooja simulator. Results show a high detection accuracy and low false positive rates.

## Introduction

Nowadays, the deployment of the Internet of Things (IoT) where many objects are connected to the Internet cloud services becomes highly recommended in many applications in various sectors. A highly important concept in the IoT is Wireless Sensor Networks or WSNs where end nodes rely on sensors that can collect data from the environment to ensure tasks such as surveillance or monitoring for wide areas [7]. This capability made the birth to the notion of smart infrastructures such as smart metering systems, smart grid or smart hospitals. In such infrastructures, end devices collecting data are connected to intermediate nodes that forward data in order to reach border routers using routing protocols. These end nodes are in general limited in terms of computational resources, battery and memory capacities. Also, their number is growing exponentially. Therefore, new protocols are proposed under the IoT paradigm to optimize energy consumption and computations. Two of these protocols are considered the de facto protocols for the Internet of Things (IoT): RPL (Routing Protocol for Low Power Lossy Network) and 6LoWPAN (IPv6 over Low Power Wireless Private Area Network). These protocols are designed for constrained devices in recent IoT applications. Routing is a key part of the IPv6 stack that remains to be specified for 6LowPan networks [6]. RPL provides a mechanism whereby multipoint-to-point traffic from devices inside the Low-Power and Lossy-Networks (LLNs) towards a central control point as well as point-to-multipoint traffic from the central control point to the device inside the LLN are supported [8, 9]. RPL involves many concepts that make it a flexible protocol, but also rather complex [10]:

- DODAG (Destination Oriented Directed Acyclic Graph): a topology similar to a tree to optimize routes between sink and other nodes for both the collect and distribute data traffics. Each node within the network has an assigned rank, which increases as the teals move away from the root node. The nodes resend packets using the lowest range as the route selection criteria.
- DIS (DODAG Information Solicitation): used to solicit a DODAG information object from RPL nodes.
- DIO (DODAG Information Object): used to construct, maintain the DODAG and to periodically refresh the information of the nodes on the topology of the network.
- DAO (DODAG Advertisment Object): used by nodes to propagate destination information upward along the DODAG in order to update the information of their parents.

With the enormous number of devices that are now connected to the Internet, a new solution was proposed: 6LowPan a lightweight protocol that defines how to run IP version 6 (IPv6) over low data rate, low power, small footprint radio networks as typified by the IEEE 802.15.4 radio [11]. In smart infrastructures, the huge amount of sensitive data exchanged among these modules and throughout radio interfaces need to be protected. Therefore, detecting any network or device breach becomes a high priority challenge for researchers due to resource constraints for devices (low processing power, battery power and memory size). Rank attack is one of the most known RPL attacks where the attacker attracts other nodes to establish routes through it by advertising false rank. This way, intruders collect all the data that pass in the network [12].

For this reason, developing specific security solutions for IoT is essential to let users catch all opportunities it offers. One of defense lines designed for detecting attackers is Intrusion Detection Systems [13] (IDS). In this paper, we propose a centralized anomaly-based IDS for smart infrastructures. We chose O-SVM (One class Support Vector Machines) algorithm for its low energy consuming compared to other machine learning algorithms for Wireless sensor network (WSN) [20].

As a use case, we are interested in smart hospital infrastructures. Such hospitals have a wide range of resources that are essential to maintain their operations, patients, employees and the building itself [1, 2] safety such as follow:

- Remote care assets: medical equipment for tele-monitoring and tele-diagnosis.
- Networked medical devices: wearable mobile devices (heartbeat bracelet, wireless temperature counters, glucose measuring devices...) or an equipment installed to collect health service related data.
- Networking equipment: standards equipment providing connectivity between different equipment (transmission medium, router, gateway...).
- Data: for both clinical and patient data, and staff data, which considered the most critical asset stored in huge datasets or private clouds.
- Building and facilities: the sensors are distributed in the hospital building that monitor the patient safety (temperature sensor for patient room and operation theater, gas sensor are among used sensors).

We target a common IoT architecture that can be considered for smart hospitals. In such architecture, there are mainly three type of components:

**Sensing Node:** composed of remote care asset, network medical device and different sensors. These sensors will send different type of data and information (patient and staff data, medical equipment status...). They are linked to micro-controllers and radio modules to transmit these data to the processing unit [3].

**Edge Router:** an edge router or border router is a specialized router residing at the edge or boundary of a network. This node ensures the connectivity of its network with external networks; a wide area network or the Internet. An edge router uses an external border gateway protocol, which is used extensively over the Internet to provide connectivity with remote networks. Instead of providing communication with an internal network, which the core router already manages, a gateway may provide communication with different networks and autonomous systems [4].

**Interface Module and Database:** this module is the terminal of the network containing all the collected data from different nodes of the network and analyze those information in order to ensure the safety of patient and improve the healthcare system.

**Fig. 1.**
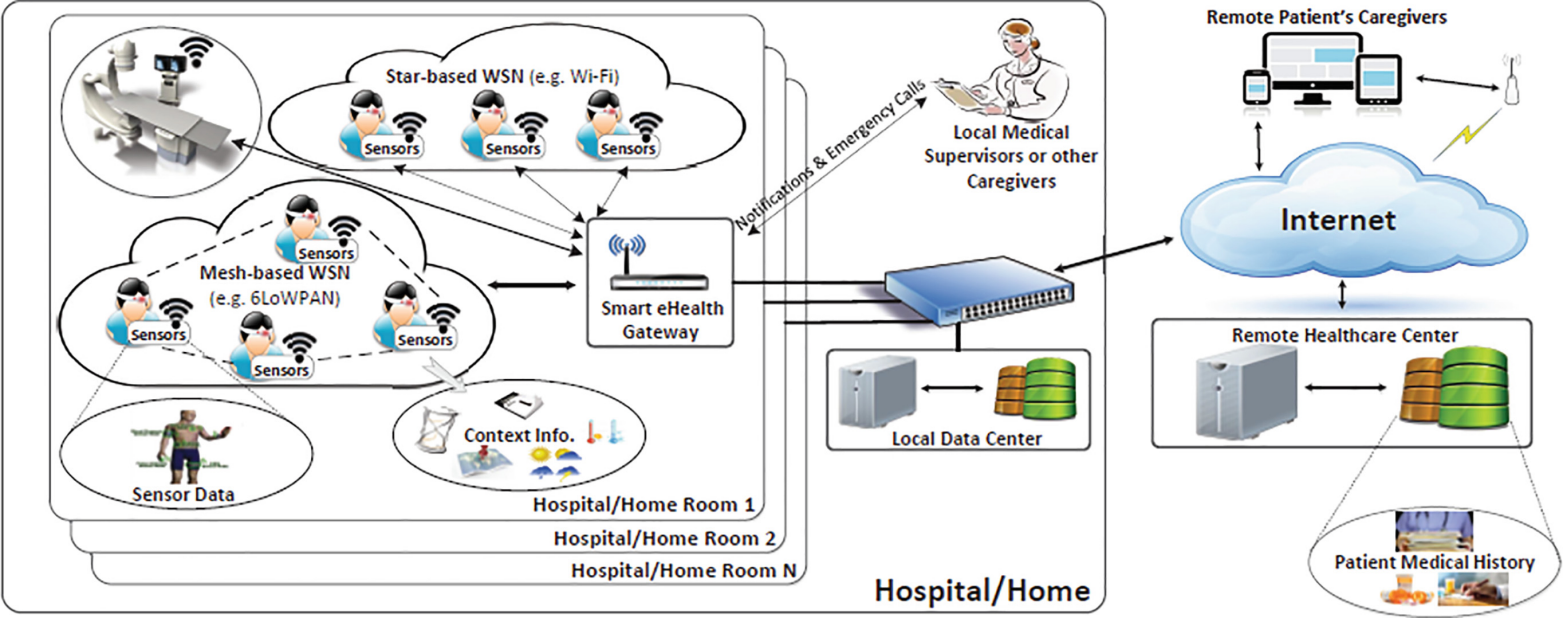
Smart hospital assets.

Figure 1 [5], presents the typical IoT e-health architecture, where sensors are distributed (medical equipment,room sensors and others) and send data to the IoT gateway. In one hand, this gives the opportunity to medical supervisor to control the patient health status. In the other hand, this data will be saved into databases for more analysis.

The rest of the paper is structured as follows. Section 2 presents the related work. Section 3 presents the Rank attack scenario. Section 4 presents our proposed approach. Section 5 presents our main results and Sect. 6 concludes the paper and presents its perspectives.

## Related Work

RPL protocol security especially in the healthcare domain is a crucial aspect for preserving personnel data. Nodes rank is an important parameter for an RPL network. It can be used for route optimization, loop prevention, and topology maintenance. In fact, the rank attack can decrease the network performance in terms of packet delivery rate (PDR) to almost 60% [23]. There were different proposed solutions to detect and mitigate RPL attacks such as rank authentication mechanism to avoid false announced ranks by using cryptography technique which was proposed in [24]. However, this technique is not very efficient because of its high computational cost and energy consumption. Authors in [25] propose a monitoring node (MN) based scheme but it is also not efficient because using a large network of MNs causes a communication overhead. In [26], authors propose the IDS called “SVELTE” that can only be used for detection of simple rank attack and has high false alarm rate. A host-based IDS was proposed in [27]. The IDS uses a probabilistic scheme but it is discouraged by RFC6550 for resource constrained networks. Routing Choice “RC” was proposed by Zhag et al. [28]. It is not directly related to the rank attack but it is based on false preferred parent selection. It has a high communication overhead in RPL networks. Trusted platform module (TPM) was proposed by Seeber et al. [29]. It introduces an overlay network of TPM nodes for detection of network attacks. SecureRPL (SRPL) [30] technique prevents RPL network from Rank attack, however it is characterized by a high energy consumption. Therefore, anomaly based solutions using machine learning permit a more efficient detection. Authors of [22] compared several unsupervised machine learning approaches based on local outlier factor, near neighbors, Mahalanobis distance and SVMs for intrusion detection. Their experiments showed that O-SVM is the most appropriate technique to detect selective forwarding and jamming attacks. Actually, we rely on these results in our choice of O-SVM.

## Rank Attack Scenario

**Fig. 2.**
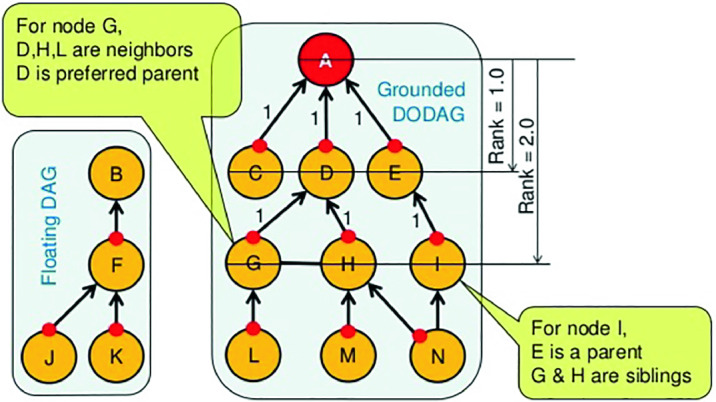
The rank in IoT network.

Rank attack is one of well known attacks against the routing protocol for low power and lossy networks (RPL) protocol in the network layer of the Internet of Things. The rank in RPL protocol as shown in Fig. 2 is the physical position of the node with respect to the border router and neighbor nodes [12].

**Fig. 3.**
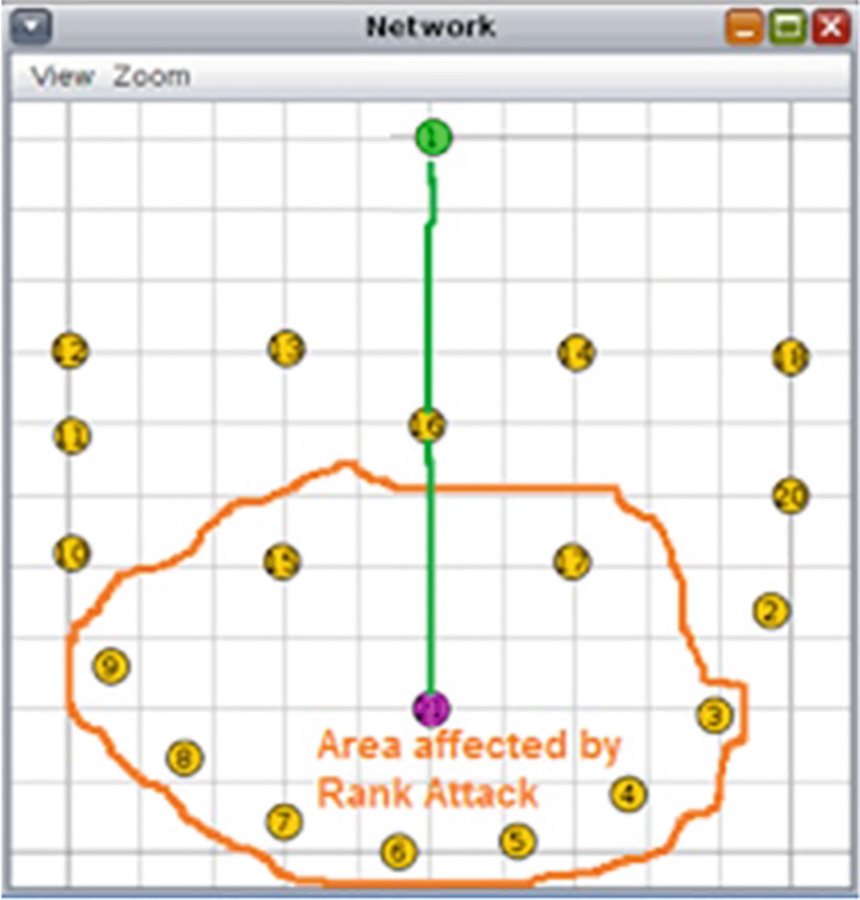
Rank attack.

Since our network is dynamic due to the mobility of its nodes (sensor moving with patient...), the RPL protocol periodically reformulates the DODAG. As shown in Fig. 3, an attacker may insert a malicious mote into the network to attract other nodes to establish routes through it by advertising false ranks while the reformulation of the DODAG is done [14].

**Fig. 4.**
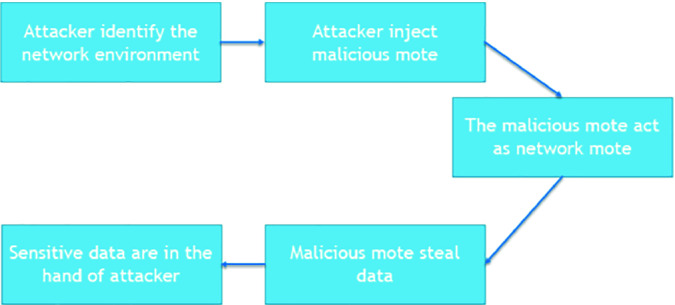
The rank attack scenario.

By default, RPL has the security mechanisms to mitigate the external attacks but it can not mitigate the internal attacks efficiently. In that case, the rank attack is considered one of dangerous attacks in dynamic IoT networks since the attacker controls an existing node (being one of the internal attack that can affect the RPL) in the DODAG or he can identify the network and insert his own malicious node and that node will act as the attack node as shown in Fig. 4.

## Proposed Approach

The key features required for our solution are to be adaptive, lightweight, and able to learn from the past. We design an IoT IDS and we implement and evaluate it as authors did in [18, 20].

**Placement Choice:** one of the important decision in intrusion detection is the placement of the IDS in the network. We use a centralized approach by installing the IDS at the border router. Therefore, it can analyze all the packets that pass through it. The choice of the centralized IDS was done to avoid the placement of IDS modules in constrained devices which requires more storage and processing capabilities [15, 16]. However, theses devices have limited resources.

**Fig. 5.**
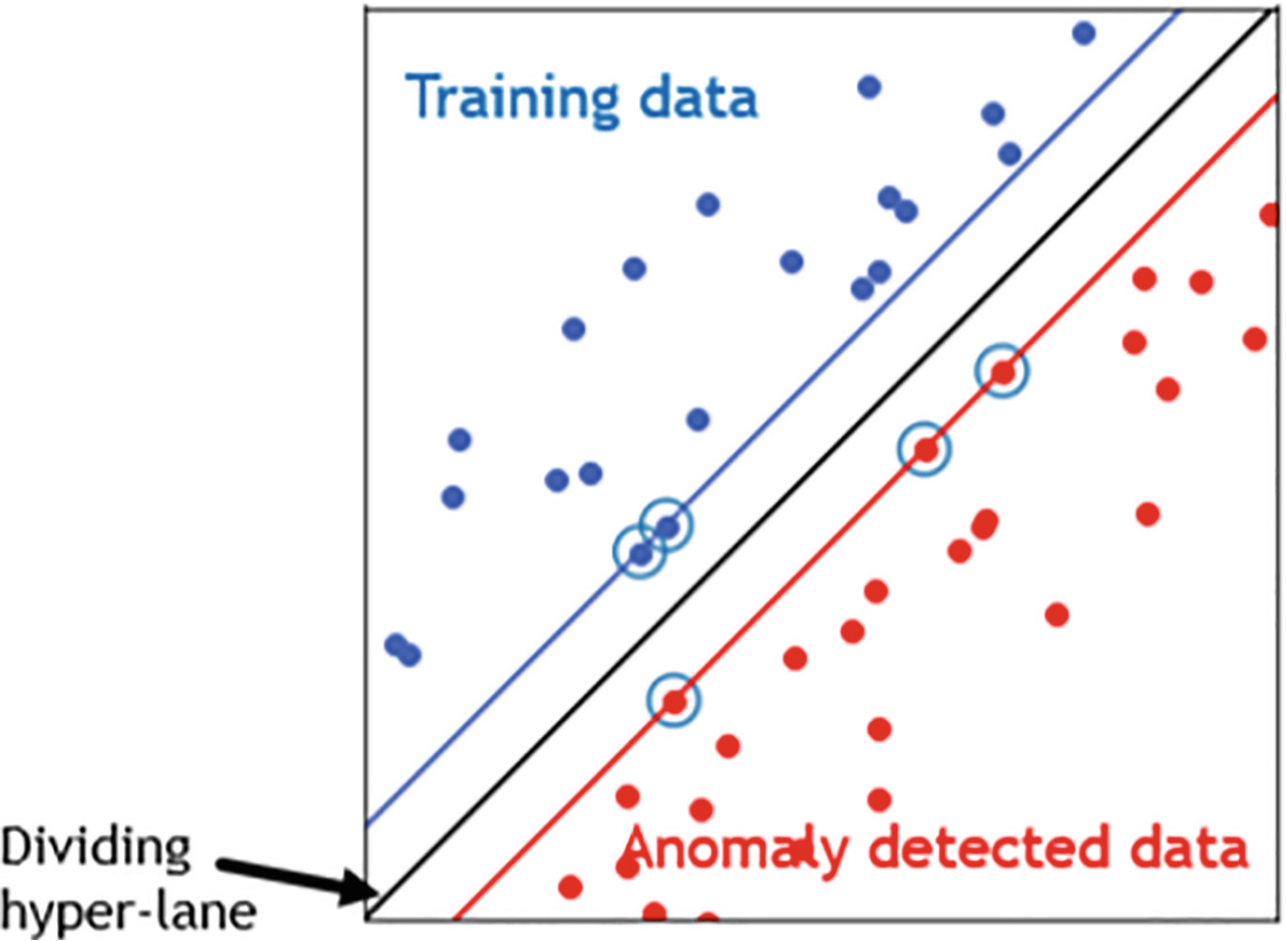
SVM classification.

**Detection Method Choice:** An intrusion detection system (IDS) is a tool or mechanism to detect attacks against a system or a network by analyzing the activity in the network or in the system itself. Once an attack is detected an IDS may log information about it and/or report an alarm [15, 16]. Broadly speaking, we aim to choose the anomaly based detection mechanisms: it tries to detect anomalies in the system by determining the ordinary behavior and using it as baseline. Any deviations from that baseline is considered as an anomaly. This technique have the ability to detect almost any attack and adapt to new environments. We chose Support Vector Machines (SVM) as an anomaly based machine learning technique. It is a discriminating classifier formally defined by a separating hyper-lane. Given labeled training data (supervised learning), the algorithm outputs an optimal hyper-lane which categorizes new examples. In two dimensional space this hyper-lane is a line dividing a plane in two parts where each class lays in either side. It uses a mathematical function named the kernel to reformulate data. After these transformations, it defines an optimal borderline between the labels. Mainly, it does some extremely complex data transformations to find a solution how to separate the data based on the labels or outputs defined. The concept of SVM learning approach is based on the definition of the optimal separating hyper-plane (Fig. 5) [21] which maximizes the margin of the training data [17, 18]. The choice of this machine learning algorithm refers to one important point, it works well with the structured data as tables of values compared to other algorithms.

We implement the IDS in the smart IoT gateway shown in Fig. 1.

## IDS Solution and Results

To investigate the effectiveness of our proposed IDS, we implement three scenarios of rank attack using Contiki-Cooja simulator [19]. We assess how our IDS module can detect them. We present next the simulation setup, evaluation metrics, and we discuss the results achieved.

### Simulation Setup

**Fig. 6.**
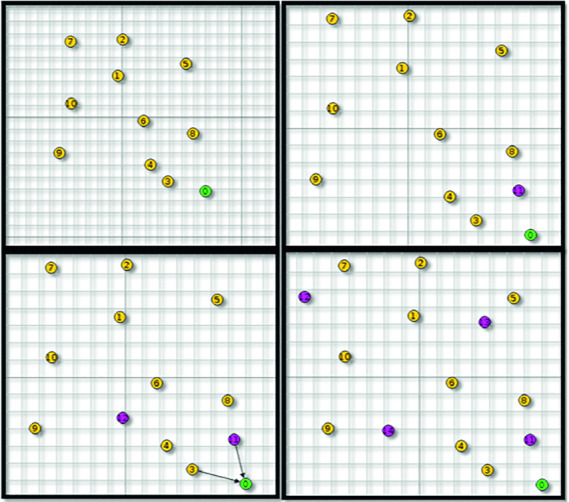
Simulation topology (Color figure online)

Our simulation scenario consists of a total 11 motes spread across an area of $200 \times 200$ m (Simulation of area of hospital where different sensors are placed in every area to control the patient rooms). The topology is shown in Fig. 6 using four scenarios. There is one sink (mote ID:0 with green dot) and 10 senders (yellow motes from ID:1 to ID:10). Every mote sends packet to the sink at the rate of 1 packet every 1 min. We implement the centralized anomaly based IDS at the root mote or the sink and we collect and analyze network data as shown in Fig. 6. We inject malicious motes (purple colour) in a random position. Table 1 summarizes the used simulation parameters.

We run four simulation scenarios for 1 h (Fig. 6):

- scenario 1: IoT network without malicious motes.
- scenario 2: IoT network with 1 randomly placed malicious mote.
- scenario 3: IoT network with 2 randomly placed malicious motes.
- scenario 4: IoT network with 4 randomly placed malicious motes.

**Table 1.** Simulation parameters

Parameter	Value
Platform	Cooja Contiki 3.0
Number of nodes	10 senders, 1 sink
Topology	Star
Area	200 m
Sending rate	1 packet/minute
Simulation run time	1 h
Number of attackers	1, 2 and then 4

### Evaluation Parameter

To evaluate the accuracy of the proposed IDS, we rely on the energy consumption parameter. We collect power tracking data per mote in terms of radio ON energy, radio transmission TX energy, radio reception RX energy and radio interfered INT energy. In order to calculate this metrics we used the formula [31] (Eq. 1, Table 2) as follow :

1 $$\begin{aligned} Energy(mJ)&= (transmit * 19.5 mA + listen * 21.8 mA \nonumber \\&+ CPU * 1.8 mA + LPM * 0.0545 mA)\nonumber \\&* 3V/4096 * 8\\ Power(mW)&= \frac{Eneregy(mJ)}{Time(s)}\nonumber \end{aligned}$$

**Table 2.** Equation parameters description

Variables	Meaning
LPM	Power consumption parameter that indicates the power used when in sleep condition
CPU	Power parameter that indicates the level of node processing
Transmit	Parameter related with node communication while transmitting
Listen	Parameter related with node communication while receiving

### Power Tracking per Mote for Each Simulation

**Fig. 7.**
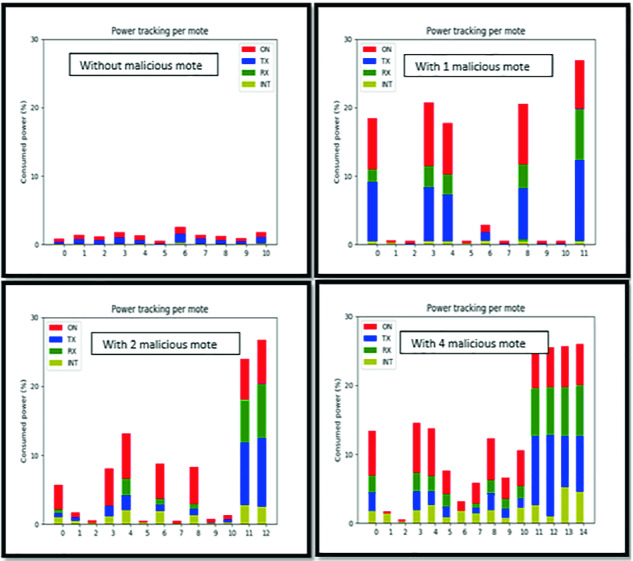
Power tracking per each mote

**Fig. 8.**
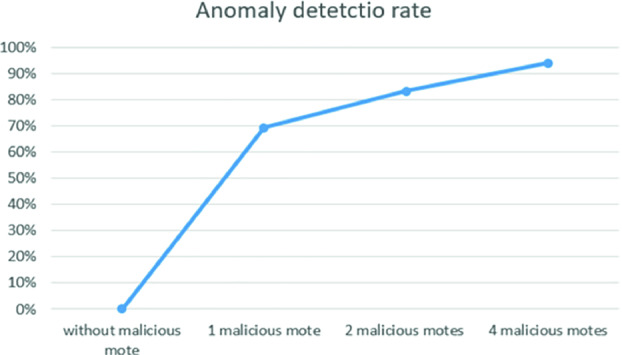
Evolution of anomaly detection rate

We used data containing 1000 instances of consumed energy values for each node in the network. Figure 7 depicts the evolution of power tracking of each node in the four scenarios:

- scenario 1: when we have a normal behavior in the network, all the sensors show a regular energy consumption in terms of receiving (node 0) and sending (nodes from 1 to 10). We use this simulation to collect the training data for the proposed IDS.
- scenario 2, 3 and 4: for those scenarios, we have a high sending values for the malicious motes. This is explained by the fact that when a malicious mote joins the network, it asks the other motes to recreate the DODAG tree and also to send data that they have, in order to steal as much data as it can. That is why it have a high receiving values too. The other motes do not distinguish that this is a malicious mote, therefore they recreate the DODAG tree, and send their information through the malicious node. We used the first simulation scenario as dataset for our IDS, describing the normal behavior of the network. This 1 h information was enough to detect the malicious activities of the rank attack. Meanwhile, each time we add a malicious mote, the anomaly detection rate increases as shown in Fig. 8.

In each simulation of malicious mote, the proposed IDS indicates the anomaly detection ratio which increases each time while adding another malicious mote. This aims to determine the impact of the number malicious motes compared to normal behavior of the system.

## Conclusion

In this paper, we propose an intrusion detection system “IDS” for smart hospital infrastructure data protection. The chosen IDS is centralized and anomaly based using a machine learning algorithm OSVM. Simulation results show the efficiency of the approach by a high detection accuracy which is more precise when the number of malicious nodes increases. As future work, we are interested in developing a machine learning based IDS for more RPL attacks detection. Furthermore, we aim to extend this solution to anomaly detection in IoT systems composed not only of WSN networks but also of cloud-based services.
